# Vitamin D in individuals before onset of rheumatoid arthritis - relation to vitamin D binding protein and its associated genetic variants

**DOI:** 10.1186/s41927-018-0033-8

**Published:** 2018-09-12

**Authors:** Mikael Brink, Linda Johansson, Evelina Nygren, Lisbeth Ärlestig, Johan Hultdin, Solbritt Rantapää-Dahlqvist

**Affiliations:** 1 Department of Public Health and Clinical Medicine/ Rheumatology, Umeå University, SE-90185 Umeå, Sweden; 2 Department of Medical Biosciences, Clinical Chemistry, Umeå University, Umeå, Sweden

**Keywords:** Rheumatoid arthritis, Vitamin D, Vitamin D binding protein, Pre-symptomatic individuals, Case-control study

## Abstract

**Background:**

Vitamin D has been implicated as being involved in the aetio-pathogenesis of several autoimmune diseases including rheumatoid arthritis (RA). Previous studies present contradictory results. Vitamin D binding protein (DBP), the major transport protein, is also involved in various inflammatory processes. The aim of this study was to investigate the relationship between circulating levels of 25-hydroxyvitamin D [25(OH) D], DBP and polymorphisms in group-specific component (GC) in pre-symptomatic individuals and matched controls within prospective cohorts of the Northern Sweden.

**Methods:**

Blood samples donated to the Medical Biobank prior to the onset of symptoms of RA (*n* = 515, mean [SD] time before the onset of symptoms 6.2 [9.3] years) and from matched (2:1) population-based controls (*n* = 267) were used. Plasma 25(OH) vitamin D levels were analyzed using liquid chromatography tandem-mass spectrometry and DBP levels were analyzed using enzyme-linked immunosorbent assay. GC polymorphisms (rs4588 and rs7041) were analyzed with TaqMan assays (Applied Biosystems).

**Results:**

Levels of 25(OH) D or DBP were not statistically different between pre-symptomatic individuals and controls in a crude, or a multiple-adjusted logistic regression model. However, an increased risk for future RA was found in females of DBP (OR 1.014 [95%CI 1.001–1.028]) per 10 mg/L adjusted for carriage of the minor allele of rs4588, in a multiple-adjusted model (*p* < 0.05).

**Conclusions:**

This study indicated that vitamin D is not associated with the future risk of RA although increasing levels of DBP were however, associated with an increased risk of disease in females carrying the minor allele of a DBP encoding SNP.

**Electronic supplementary material:**

The online version of this article (10.1186/s41927-018-0033-8) contains supplementary material, which is available to authorized users.

## Background

Vitamin D, a hormone that has, due to its immunomodulatory properties, been implicated as an aetiological factor of autoimmune diseases, including rheumatoid arthritis (RA) [1]. Furthermore, vitamin D has been shown to influence several immune cells, including T-, B- and dendritic cells [1, 2]. Vitamin D_3_ is produced in the skin as well as taken up from dietary sources, whilst vitamin D_2_ is found mainly in supplements. In circulation vitamin D binding protein (DBP), also known as group-specific component (GC)-globulin, is the major protein transporter of vitamin D, accounting for transportation of 85–90% of all vitamin D in plasma, with the remaining fraction 10–15% bound to albumin and less than 1% in an unbound form [3]. DPB is present in a high (20-fold) molar excess compared with vitamin D. At most, only 5% of DBP binds vitamin D. DBP has been linked to several steps involving the inflammatory processes, e.g. involved in the complement-mediated tissue recruitment of neutrophils and by conversion to DBP-macrophage activating factor is a naturally occurring protein with the ability to activate macrophages. Furthermore DBP has been suggested to influence T cell response [3]. In the liver, vitamin D is converted to 25-hydroxyvitamin D [25(OH)D]. It is biologically inactive and must be activated via enzymes in the kidneys to 1,25-dihydroxyvitamin D [1,25(OH)_2_D]. 1,25(OH)_2_D is then taken up by target cells and exerts its action by binding to the intracellular vitamin D receptor (VDR) to form a complex together and functions to alter gene transcription [4]. Polymorphisms of the *GC* gene (the gene encoding DBP), where the most consistent findings are for the single nucleotide polymorphisms (SNPs), rs4588 and rs7041, are major genetic contributors of variations in 25(OH) D levels, also causing significant variations in DBP levels [5, 6].

Rheumatoid arthritis is an autoimmune disease primarily affecting the joints of hands and feet with both environmental and genetic risk factors contributing to disease susceptibility [7]. Previous studies investigating the relationship between future RA and assessed vitamin D status, based on food frequency questionnaires, have reached different results, such as a suggestive lower risk of developing RA following a higher vitamin D intake or no association with vitamin D intake and risk for RA, respectively [8–10]. By measuring circulating vitamin D levels in serum before the clinical onset of RA symptoms, one study found no difference in vitamin D levels in pre-symptomatic individuals compared with controls [11], whilst another study demonstrated that pre-symptomatic individuals had a 20% decreased risk per unit of 25(OH)D, in those sampled 3 months to < 4 years before onset of symptoms [12]. The fact that the association between RA and vitamin D varies between studies could, at least partly, be the result of different methods of determining the vitamin D status, where the LC-MS/MS is considered the gold standard [13–15].

The objective with our study was to investigate the 25(OH) D and DBP levels, and taking new confounding factors, such as the polymorphisms of the *GC* gene, into account, with the aim of understanding the potential risk factors for future development of RA using a nested case-control study design within the Medical Biobank of Northern Sweden.

## Methods

### Study population

A nested case-control study was conducted using individuals included in population surveys within the Northern Sweden Health and Disease Study (NSHDS), with the catchment area being above latitude 63°N. The criteria for recruitment, and the collection and storage of blood samples, have previously been described in detail [16]. The cohorts studied derived from the Medical Biobank (Västerbotten intervention program [VIP], monitoring trends and determinants in cardiovascular disease [MONICA] and The Mammography screening project) are population-based, and all adult individuals residing in the county of Västerbotten are continuously invited to participate. The registers from the Medical Biobank were co- analyzed with the registers of patients fulfilling the 1987 American Rheumatism Association classification criteria for RA [17] at the Department of Rheumatology, University Hospital, in Umeå, Sweden, with a known date for the onset of symptoms to identify individuals having donated blood samples before symptom onset. This co-analysis of registers identified 515 individuals (150 males/365 females), 420 (81.6%) from the VIP, 55 (10.7%) from the Mammography screening project and 40 (7.8%) from MONICA project, respectively who had participated in these projects prior to the onset of symptoms (referred to as “pre-symptomatic individual” or case). Control subjects (*n* = 267) (79 males/ 188 female) were randomly identified from the same cohorts (226 (84.6%) from the VIP, 31 (11.6%) from the Mammography screening project and 10 (3.7%) from MONICA, respectively, and matched (2:1) for age, sex and date of blood sampling. Body Mass Index (BMI, kg/m^2^) was calculated from the anthropometric measures of weight and height, available for the participants in MONICA and VIP studies. Participants from MONICA and VIP also completed a health questionnaire regarding socioeconomic and demographic status, educational level (analyzed as no academic education/university vs. academic education/university) self-reported health and lifestyle (e.g., smoking habits and intake of multivitamin supplements). Sampling time of the year was defined as light (April–September) or dark (October to March) season.

### 25(OH) D measurement

Plasma levels of cholecalciferol (25(OH)D_3_) and ergocalciferol (25(OH)D_2_) were determined using liquid chromatography-tandem mass spectrometry (LC-MS/MS) using an Agilent 1200/Sciex API 4000, (Agilent Technologies Inc., Santa Clara, CA, USA) and a Shimadzu Nexera/Sciex QTrap 5500 (Shimadzu Corporation, Kyoto, Japan). For calibration the 6PLUS1® Multilevel Serum Calibrator Set 25-OH-Vitamin D_3_/D_2_ (Order no.: 62039) was used, and as internal control the 25-OH-Vitamin D_3_/D_2_ Serum Control Bi-Level (Order no.: 0028), both from Chromsystems Instruments & Chemicals GMBH (Gräfelfing, Germany). To ensure analytical quality, external controls from DEQAS (www.deqas.org) were used with values assigned by the NIST Reference Measurement Procedure. The total coefficients of variation for 25(OH)D_3_ were 13.9%, and 12.6% at levels of 33.61 and 133.63 nmol/L, respectively, in this study. The corresponding values of 25(OH)D_2_ were 13.7%, and 11.9% at levels of 31.85, and 128.17 nmol/L, respectively. The lower limit of quantification (LOQ) for Vitamin D2 and Vitamin D3 was 10 nmol/L. Total Vitamin D was calculated by adding Vitamin D2 and Vitamin D3 levels. In the event of a value below the LOQ that value was considered to be zero.

### Vitamin D binding protein (DBP) assay

DBP concentrations derived from EDTA-plasma samples were analyzed using Quantikine ELISA Human Vitamin D BP kit, DVDBP0 according to the manufacturers’ instructions (R&D Systems, Minneapolis, MN, USA). Inter-assay coefficient of variation was 5.6% at 243 mg/L.

The molecular weight for DBP (58.0 kDa) was used for calculation of the molar ratio 25(OH)D:DBP (×10^3^). This was used as a proxy for free circulating 25(OH)D [18]. Calculation of total 25(OH)D:DBP ratio was calculated according to Powe et al. [19].

### Genotyping

Genomic DNA was extracted from buffy-coat cells using standard protocols. The two SNPs rs7041 and rs4588 were genotyped using commercially available TaqMan assays (Applied Biosystems) according to the manufacturer’s instructions. *GC* haplotypes were found in four variants CG (in literature also referred as GC-1S), AT (GC-2), CT (GC-1F) and AG (GC-3). Since the AG haplotype was only found in one individual (a pre-symptomatic female) this individual was excluded from further analyses including haplotype/diplotype combinations. The distribution of both polymorphisms was in Hardy-Weinberg equilibrium in both cases and controls.

### Statistics

Statistical calculations were performed using SPSS for Windows version 24.0 (IBM Corp., NY, USA). The associations with future RA were analyzed in logistic regression models with calculated odds ratios (ORs) and 95% confidence intervals (CIs). All *p* values are two-sided and *p* < 0.05 was considered statistically significant. Genotype and allele frequencies as well as odds ratios and their 95% confidence intervals (95% CIs) were calculated. Comparisons of genotype and allele frequencies were performed using a chi-squared test. Hardy Weinberg proportions were calculated using Haploview. Correlations between two continuous variables were analyzed with Spearman rank correlation test. Cox proportional hazard regression analyses were performed with case/control as the dependent variable and time to event (i.e. symptom onset) was set to the same value within the matching case-control pair.

Pre-study power calculation was based on t-test, using Sample Size Calculator (http://clincalc.com/). Due to the scarcity of prospective studies on pre-RA individuals we used data from a recent Japanese retrospective study [20], with mean levels of 25(OH)D of 16.9 and 19.5 ng/mL for patients with RA and controls, respectively. This corresponds to a difference of 15% between the groups. As a conservative approach sigma (common standard deviation) was set as 10. Two-tailed significance test at an alpha level of 0.05 and with case/control ratio 2:1 yielded a statistical power of 80% for 348 cases and 174 controls. In a study from a neighboring country, Estonia [21] reported a 23% difference during winter. Based on this calculation, 515 cases and 267 controls were assessed as sufficient.

## Results

### Characteristics of the participants and gene distributions

The baseline characteristics of the pre-symptomatic individuals and controls are presented in Table 1. The pre-symptomatic individuals were more frequently smokers (*p* < 0.001), had a higher BMI and lower educational level compared with controls (*p* < 0.05) (Table 1). The season (dark/light) at time of sample collection was similar between cases and controls (Table 1).

**Table 1:** Characteristics of all included controls, and pre-symptomatic individuals, also separated by gender

	All			Male			Female		
	Controls (*n* = 267)	Pre-symptomatic individuals (*n* = 515)	*p* ^a^	Controls (*n* = 79)	Pre-symptomatic individuals (*n* = 150)	*p* ^a^	Controls (*n* = 188)	Pre-symptomatic individuals (*n* = 365)	*p* ^a^
Gender, female n (%)	188 (70.4)	365 (70.9)	0.89	–	–	–	–	–	–
Age at sample donation, years, mean (SD)	53.3 (9.3)	52.2 (9.4)	0.14	52.3 (8.7)	51.6 (9.3)	0.59	53.7 (9.5)	52.5 (9.5)	0.15
Time between sampling and onset of symptoms, years (SD)	–	6.2 (4.3)	–	–	6.3 (4.2)	–	–	6.2 (4.3)	–
BMI, mean (S.D.)	25.4 (3.8)	26.3 (4.3)	**0.01**	25.8 (2.6)	26.9 (3.9)	**0.03**	25.2 (4.2)	26 (4.4)	0.10
Ever smoker, n (%)	103/242 (42.6)	324/514 (63.0)	**< 0.001**	42/76 (55.3)	103/150 (68.7)	**< 0.05**	61/166 (36.7)	221/364 (60.7)	**< 0.001**
Education level^b^, n (%)	173/224 (77.2)	377/453 (83.2)	0.06	55/72 (76.4)	133/149 (89.3)	**0.01**	118/152 (77.6)	244/304 (80.3)	0.51
Season at time of sample collection^c^, n (%)	124/267 (46.4)	266/515 (51.7)	0.61	48/79 (60.8)	75/150 (50.0)	0.12	93/188 (49.5)	174/365 (47.7)	0.68
Total 25(OH)D nmol/L, mean (SD)	54.5 (17.4)	53.8 (17.7)	0.56	53.7 (17.7)	55.5 (17.6)	0.45	54.9 (17.3)	53.1 (17.7)	0.24
DBP mg/L, mean (SD)	278 (117.8)	279.2 (117.6)	0.89	265.4 (122.8)	249.1 (111.3)	0.31	283.3 (115.5)	291.6 (118)	0.43
Total 25(OH)D:DBP molar ratio^d^, mean (SD)	14.1 (8.8)	13.9 (9.8)	0.77	15.4 (10.4)	16.3 (10.6)	0.51	13.5 (8)	12.9 (9.2)	0.39

^a^Calculated with Mann-Whitney U test for continuous variables or Pearson Chi-Square for dichotomous variables

^b^Presented as having no academic education/university

^c^Presented as light time of year, including sampling time between March and September

^d^Proxy for free vitamin D concentration

The significant p-values are shown in bold

The genotype, allele frequencies for two polymorphisms (rs4588 and rs7041) in the *GC* gene, and haplotype and diplotype frequencies, defined by the combination of rs4588 and rs7041 alleles did not differ between cases and controls (Additional file 1: Table S1).

### Total and free 25-Hydroxyvitamin D levels in pre-symptomatic individuals and controls

Levels of total 25(OH) D did not differ between pre-symptomatic individuals and controls (mean [SD] 53.9 [17.7] nmol/L vs. 54.5 [17.4] nmol/L) (Table 1), or after adjustments for BMI, season and age at the time of sample collection, ever smoking, and educational level (data not shown). Levels of free 25(OH) D did not differ between pre-symptomatic individuals and controls (mean [SD] 14.1 [8.8] nmol/L vs. 13.9 [9.8] nmol/L), or after the aforementioned adjustments (Table 1). Due to a positive correlation between 25(OH) D levels and DBP levels (*r* = 0.183, *p* < 0.001) further adjustment for DBP levels did not alter the non-significant results. Adjusting for the genotypes, the alleles and for *GC* haplotypes or *GC* diplotypes, respectively, did not influence the models analysing 25(OH) D levels (Additional file 1: Table S2).

Stratification for the predating time before symptom onset, namely between 3 months to < 4 years and ≥ 4 years did not yield any significant differences between cases and controls in respect of 25(OH) D concentration, with the same adjustments. Additional adjustment for DBP level did not change the results further (data not shown). The levels of 25(OH) D did not vary significantly between light and dark season of the year in pre-symptomatic individuals (mean [SD] 55.1[18.4] and 52.5 [17]) or controls (mean [SD] 54.2[18.4] and 54.8 (16.6), respectively. No difference in levels of 25(OH) D was found in pre-symptomatic individuals or controls who stated intake of multivitamin supplements within the last 14 days (*n* = 38 pre-symptomatic individuals and *n* = 28 controls) or within the last year (*n* = 61 pre-symptomatic individuals and *n* = 42 controls).

### DBP levels in pre-symptomatic individuals and controls

DBP concentrations were similar between all pre-symptomatic individuals and controls (279.2 (117.6) mg/L vs. 278 (117.7) mg/L), although stratification for sex revealed significantly higher levels in females (*p* < 0.001). After the afore mentioned adjustments no significant difference was found in DBP levels when comparing pre-symptomatic individuals and controls stratified for sex. A significant relationship was found between increasing levels of DBP and being pre-symptomatic female individuals when adjusting for 25(OH) D levels and the minor allele (A) of SNP rs4588 besides BMI (*p* < 0.05) (Table 2). Adjustment for combinations of these alleles as haplotypes or diplotypes (Additional file 1: Table S2) did not show any difference in DBP levels between pre-symptomatic individuals and controls (data not shown).

**Table 2:** Association between pre-symptomatic individuals and controls separated by gender in a univariable and multivariable logistic regression analysis

	Male				Female			
	Univariable		Multivariable^†^		Univariable		Multivariable^†^	
	OR (95%CI)	*p*-value	OR (95%CI)	*p*-value	OR (95%CI)	*p*-value	OR (95%CI)	*p*-value
Total 25(OH) D, (nmol/L)	1.01 (0.99–1.02)	0.451	1 (0.99–1.02)	0.524	0.99 (0.98–1)	0.243	0.99 (0.99–1)	0.184
DBP per 1 mg/L	1.00 (1.00–1.00)	0.310	1.00 (1.00–1.00)	0.631	1.00 (1.00–1.00)	0.433	1.001 (1.000–1.003)	**0.034**
DBP, per 10 mg/L	0.988 (0.965–1.011)	0.310	0.995 (0.976–1.015)	0.631	1.006 (0.991–1.021)	0.433	1.014 (1.001–1.028)	**0.034**
Allele A^1^	0.70 (0.46–1.08)	0.104	0.68 (0.41–1.12)	0.126	1.22 (0.91–1.62)	0.182	1.27 (0.9–1.79)	0.167

^1^Allele A frequency in pre-symptomatic males 0.25 and in females 0.28

†Adjusted for BMI, sampling time of year (dark/light), smoking ever, educational level (academic/ no academic), age at the time of sampling

The significant p-values are shown in bold

### Levels of vitamin D and DBP in relation to sero-positivity in pre-symptomatic individuals

Levels of 25(OH) D did not differ between anti-CCP2 or RF-IgM positive vs. negative individuals among the pre-symptomatic individuals in a logistic regression analysis with the mentioned adjustments (sex, season and age at time of sample collection, BMI and educational level) (data not shown).

When applying the same adjustments, no association was found between levels of DBP (mg/L) and anti-CCP2 or RF-IgM positivity vs. negativity in pre-symptomatic individuals (data not shown).

## Discussion

In this study, a higher level of DBP was found to be associated with an increased risk of the future development of RA in females, adjusted for rs4588 minor allele, smoking, BMI, educational level, age and sampling time of the year. We did not observe any association between total Vitamin D level or DBP level in plasma and a future risk of RA, irrespective of sex or gene polymorphism.

Conflicting results have been presented regarding the role of vitamin D and the risk of developing RA in the future, this can partly be explained by the many factors that are known to influence the circulating levels of Vitamin D in man, e.g., exposure to Ultraviolet B (UVB) light, geographical location, skin pigmentation, protective measures from sunlight and intake of Vitamin D containing nutrients [4]. Also, both vitamin D status and levels of DBP are associated with *GC* variants, and in particular those analyzed in this study, rs7041 and rs4588 [22–24]. Similar to the results of their studies, we found a gene-dosage relationship with differences between the rs7041 and rs4588 genotypes and DBP and 25(OH) D levels, showing lowest vs. highest levels in rs7041 TT vs. GG and rs4588 AA vs. CC and intermediate levels in heterozygotes, with a similar pattern in controls and pre-symptomatic individuals. In this study a positive correlation between levels of DBP and 25(OH) D, was confirmed in line with previous published studies [24, 25]. We found significantly higher DBP levels in females compared with males, as previously suggested [26, 27].

Levels of DBP have been associated with BMI, oral contraceptive use, lipid parameters, and current smoking [3], and we have adjusted for the variables BMI, educational level [academic vs. non-academic], smoking status (ever having smoked [Yes/No]), age at time of sampling and the season at the time of blood collection (Dark vs. Light). We have no information on the use of contraceptive pills by those in our cohort of cases, although the mean age of the females was 54.4 years, suggesting that a majority were in menopause.

There are two earlier studies on pre-symptomatic individuals who later developed RA based on levels of Vitamin D published. The study by Nielen et al. [11] analyzed 25(OH) D in 79 patients with RA who had previously donated blood to a blood bank, selecting one sample from each individual from time points 1 year, 2 years, and ≥ 5 years before the start of symptoms together with matched controls. They compared the proportion of 25(OH) D deficiency (20, 12.5, 10, 7.5 or 5 nmol/L as cut-off) among cases and controls without finding significant differences for any of the cutoff levels. However, no linear relationship of concentrations was investigated, nor were any other adjustments of factors with known relationships to both RA risk and 25(OH) D levels, e.g. BMI [4, 28]. Similarly, a report by Hiraki et al. did not describe any overall difference in circulating 25(OH) D levels between pre-symptomatic women and matched controls, although when stratified by time females presented an inverse linear association between 25(OH) D levels and future RA for the time group closest to symptom onset (3 months to < 4 years) in one of their two sub cohorts, which we were unable to reproduce [12]. In that published report, although a lot of confounding factors were adjusted for, levels of DBP and/ or GC-polymorphisms were not taken into account, nor were differences inherently related to the method of determining the concentration of 25(OH) D. Several publications investigating the performance of commercially available kits for 25(OH) D analysis, were radioimmunoassays (RIA) (as in the report by Hiraki et al., 2014), as well as chemiluminescent immunoassays (CLIA) and enzyme-linked immunosorbent assays (ELISA) (used in the paper by Nielen et al., 2006) have shown great inter-assay differences, especially in the concentration interval corresponding to insufficiency of vitamin D, compared with HPLC methods. This may cause misclassification when, for example, using 50 nmol/L as cutoff [14, 29]. In a previous report sero-positivity, either for ≥2 RF-isotypes or anti-CCP2 antibodies among individuals at risk of developing RA, was not found to be associated with higher 25(OH) D levels [30]. Likewise, in our study no association was found between anti-CCP2 antibodies or RF-IgM-positivity and 25(OH) D levels when adjusting for relevant confounders. Additionally, anti-CCP2 antibodies and RF-IgM positivity was not associated with DBP levels when adjusting for relevant confounders.

We acknowledge some limitations in this present study. For example, we included only one sample per individual, which may not reflect the long term vitamin D status. For analysis of DBP we used a monoclonal antibody, which may underestimate concentrations in subjects with an African ancestry, a problem not experienced in the methods using polyclonal antibodies [31] However, the population of northern Sweden is ethnically homogenous [32].

Our study had several strengths, including measurement of total 25(OH) D using HPLC, a protocol currently considered the gold standard method capable of distinguishing between D_3_ and D_2_ forms in an accredited laboratory setting. We measured levels of DBP and *GC* polymorphisms in addition to having the possibility of adjusting for possible confounding factors, BMI, education level and smoking status, factors that are associated with both outcome and exposure. By analysing DBP we were also able to calculate a proxy for free vitamin D. This was also the largest cohort of pre-symptomatic RA individuals studied to date when investigating vitamin D, with the ability to find differences for both genders.

The outcome of this study has added to knowledge about the relationship between D-vitamin status and the risk of developing RA.

## Conclusion

This study found no differences in vitamin D or DBP levels between pre-symptomatic individuals and population controls, although in a subset of females an increased risk of developing RA in individuals with elevated DBP levels was observed in a multiple adjusted logistic regression model.

## Additional file

Additional file 1: Two tables describing the distribution of *GC* polymorphisms across study groups and risk of disease development in univariable and multivariable logistic regression models stratified for haplotypes and diplotypes. (DOCX 23 kb)

## Abbreviations

25(OH) D: Vitamin D
Anti-CCP: Anti-cyclic citrullinated peptide antibodies
BMI: Body mass index
DBP: Vitamin D binding protein
ELISA: Enzyme-linked immunosorbent assay
HPLC: High pressure liquid chromatography
LC-MS/MS: Liquid chromatography, tandem mass spectrometry
LOQ: Limit of quantification
MONICA: Monitoring trends and determinants in cardiovascular disease
NSHDS: Northern Sweden health and disease study
RA: Rheumatoid arthritis
RF: Rheumatoid Factor
RIA: Radioimmunoassay
SD: Standard deviation
SNP: Single nucleotide polymorphism
VIP: Västerbotten intervention program

## References

[1] Arnson Y, Amital H, Shoenfeld Y (2007). Vitamin D and autoimmunity: new aetiological and therapeutic considerations. Ann Rheum Dis.

[2] Deluca HF, Cantorna MT (2001). Vitamin D: its role and uses in immunology. FASEB J.

[3] Delanghe JR, Speeckaert R, Speeckaert MM (2015). Behind the scenes of vitamin D binding protein: more than vitamin D binding. Best Pract Res Clin Endocrinol Metab.

[4] Holick MF (2017). The vitamin D deficiency pandemic: approaches for diagnosis, treatment and prevention. Revi Endocrine Metabol Disord.

[5] Wang TJ, Zhang F, Richards JB, Kestenbaum B, van Meurs JB, Berry D (2010). Common genetic determinants of vitamin D insufficiency: a genome-wide association study. Lancet.

[6] McGrath JJ, Saha S, Burne THJ, Eyles DW (2010). A systematic review of the association between common single nucleotide polymorphisms and 25-hydroxyvitamin D concentrations. J Steroid Biochem Mol Biol.

[7] Klareskog L, Catrina AI, Paget S (2009). Rheumatoid arthritis. Lancet.

[8] Costenbader KH, Feskanich D, Holmes M, Karlson EW, Benito-Garcia E (2008). Vitamin D intake and risks of systemic lupus erythematosus and rheumatoid arthritis in women. Ann Rheum Dis.

[9] Hiraki LT, Munger KL, Costenbader KH, Karlson EW (2012). Dietary intake of vitamin D during adolescence and risk of adult-onset systemic lupus erythematosus and rheumatoid arthritis. Arthritis Care Res (Hoboken).

[10] Merlino LA, Curtis J, Mikuls TR, Cerhan JR, Criswell LA, Saag KG (2004). Vitamin D intake is inversely associated with rheumatoid arthritis: results from the Iowa Women's health study. Arthritis Rheum.

[11] Nielen MMJ, van Schaardenburg D, Lems WF, van de Stadt RJ, de Koning MHMT, Reesink HW (2006). Vitamin D deficiency does not increase the risk of rheumatoid arthritis: comment on the article by Merlino et al. Arthritis Rheum.

[12] Hiraki LT, Arkema EV, Cui J, Malspeis S, Costenbader KH, Karlson EW (2014). Circulating 25-hydroxyvitamin D level and risk of developing rheumatoid arthritis. Rheumatology (Oxford).

[13] Lai JK, Lucas RM, Banks E, Ponsonby AL (2012). Variability in vitamin D assays impairs clinical assessment of vitamin D status. Intern Med J.

[14] Snellman G, Melhus H, Gedeborg R, Byberg L, Berglund L, Wernroth L (2010). Determining vitamin D status: a comparison between commercially available assays. PLoS One.

[15] Zerwekh JE (2008). Blood biomarkers of vitamin D status. Am J Clin Nutr.

[16] Rantapää-Dahlqvist S, de Jong BAW, Berglin E, Hallmans G, Wadell G, Stenlund H (2003). Antibodies against cyclic citrullinated peptide and IgA rheumatoid factor predict the development of rheumatoid arthritis. Arthritis Rheum.

[17] Arnett FC, Edworthy SM, Bloch DA, McShane DJ, Fries JF, Cooper NS (1988). The American rheumatism association 1987 revised criteria for the classification of rheumatoid arthritis. Arthritis Rheum.

[18] Bouillon R, Van Assche FA, Van Baelen H, Heyns W, De Moor P (1981). Influence of the vitamin D-binding protein on the serum concentration of 1,25-Dihydroxyvitamin D(3): significance of the free 1,25-Dihydroxyvitamin D(3) concentration. J Clin Invest.

[19] Powe CE, Ricciardi C, Berg AH, Erdenesanaa D, Collerone G, Ankers E (2011). Vitamin D–binding protein modifies the vitamin D–bone mineral density relationship. J Bone Miner Res.

[20] Matsumoto Y, Sugioka Y, Tada M, Okano T, Mamoto K, Inui K (2015). Relationships between serum 25-hydroxycalciferol, vitamin D intake and disease activity in patients with rheumatoid arthritis--TOMORROW study. Mod Rheumatol.

[21] Cutolo M, Otsa K, Laas K, Yprus M, Lehtme R, Secchi ME (2006). Circannual vitamin d serum levels and disease activity in rheumatoid arthritis: northern versus southern Europe. Clin Exp Rheumatol.

[22] Gozdzik A, Zhu J, Wong BYL, Fu L, Cole DEC, Parra EJ (2011). Association of vitamin D binding protein (VDBP) polymorphisms and serum 25(OH)D concentrations in a sample of young Canadian adults of different ancestry. J Steroid Biochem Mol Biol.

[23] Sinotte M, Diorio C, Berube S, Pollak M, Brisson J (2009). Genetic polymorphisms of the vitamin D binding protein and plasma concentrations of 25-hydroxyvitamin D in premenopausal women. Am J Clin Nutr.

[24] Medlej-Hashim M, Jounblat R, Hamade A, Ibrahim JN, Rizk F, Azzi G (2015). Hypovitaminosis D in a young Lebanese population: effect of GC gene polymorphisms on vitamin D and vitamin D binding protein levels. Ann Hum Genet.

[25] Lauridsen AL, Vestergaard P, Hermann AP, Brot C, Heickendorff L, Mosekilde L (2005). Plasma concentrations of 25-hydroxy-vitamin D and 1,25-dihydroxy-vitamin D are related to the phenotype of Gc (vitamin D-binding protein): a cross-sectional study on 595 early postmenopausal women. Calcif Tissue Int.

[26] Blanton D, Han Z, Bierschenk L, Linga-Reddy MV, Wang H, Clare-Salzler M (2011). Reduced serum vitamin D-binding protein levels are associated with type 1 diabetes. Diabetes.

[27] Bolland MJ, Grey AB, Ames RW, Horne AM, Mason BH, Wattie DJ (2007). Age-, gender-, and weight-related effects on levels of 25-hydroxyvitamin D are not mediated by vitamin D binding protein. Clin Endocrinol.

[28] Ljung L, Rantapaa-Dahlqvist S (2016). Abdominal obesity, gender and the risk of rheumatoid arthritis - a nested case-control study. Arthritis Res Ther.

[29] He C-S, Gleeson M, Fraser WD (2013). Measurement of circulating 25-hydroxy vitamin d using three commercial enzyme-linked immunosorbent assay kits with comparison to liquid chromatography: tandem mass spectrometry method. ISRN Nutr.

[30] Feser M, Derber LA, Deane KD, Lezotte DC, Weisman MH, Buckner JH (2009). Plasma 25,OH vitamin D concentrations are not associated with rheumatoid arthritis (RA)-related autoantibodies in individuals at elevated risk for RA. J Rheumatol.

[31] Nielson CM, Jones KS, Chun RF, Jacobs J, Wang Y, Hewison M (2016). Role of assay type in determining free 25-Hydroxyvitamin D levels in diverse populations. N Engl J Med.

[32] Humphreys K, Grankvist A, Leu M, Hall P, Liu J, Ripatti S (2011). The genetic structure of the Swedish population. PLoS One.

