# Supplementary material for: Vitamin D in individuals before onset of rheumatoid arthritis - relation to vitamin D binding protein and its associated genetic variants
Source: BMC Rheumatol. 2018 Sep 12;2:26. doi: 10.1186/s41927-018-0033-8 (PMC6390591; doi:10.1186/s41927-018-0033-8)
Supplement: Supplementary file 1 — Two tables describing the distribution of GC polymorphisms across study groups and risk of disease development in univariable and multivariable logistic regression models stratified for haplotypes and diplotypes. (DOCX 23 kb) [file 41927_2018_33_MOESM1_ESM.docx]

# Additional file 1

## **Table S1. Distribution of *GC* polymorphisms rs4588 and rs7041, haplotypes and diplotypes.**

| ***GC* SNP** | **Genotypes** | **0** | **1** | **2** | | **MAF** | **HWE** |
| --- | --- | --- | --- | --- | --- | --- | --- |
|  | **0/1/2** | **Case/ control, n(%)** | **Case/ control, n(%)** | **Case/ control, n(%)** | | **Case/ controls** | **p-value** |
| rs4588 | CC/AC/AA | 268 (52.2)/ 146 (55.7) | 212 (41.3)/ 93 (35.5) | 33 (6.4)/ 23 (8.8) | | 0.271/ 0.265 | 1.0 |
| rs7041 | TT/GT/GG | 77 (15)/ 45 (17.2) | 243 (47.4)/ 112 (42.7) | 193 (37.6)/ 105 (40.1) | | 0.387/ 0.385 | 0.373 |
| ***GC* Haplotypes** | **Haplotype** | **Frequency**  **Case / control** | **Haplotype ratio**  **Case / control** | **χ^2^** | **p-value** | | |
|  | 1S/ CG | 0.612/ 0.615 | 628:396 /322:202 | 0.002 | 0.96 | | |
|  | 2/ AT | 0.270/ 0.265 | 276:748 / 139:385 | 0.032 | 0.86 | | |
|  | 1F/ CT | 0.117/ 0.120 | 120:904 / 63:461 | 0.031 | 0.86 | | |
| ***GC* Diplotypes** | **Diplotype** | **Frequency  Case/control** | **Diplotype ratio**  **Case/ control** | **χ^2^** | **p-value** | | |
|  | 1F-1F/ CTCT | 0.02/ 0.019 | 10:512/ 5:262 | 0.002 | 0.96 | | |
|  | 1F-1S/ CGCT | 0.127/ 0.137 | 65:512/ 36:262 | 0.17 | 0.68 | | |
|  | 1F-2/ CTAT | 0.068/ 0.065 | 35:512/ 17:262 | 0.33 | 0.86 | | |
|  | 1S-1S/ CGCG | 0.377/ 0.401 | 193:512/ 105:262 | 0.42 | 0.52 | | |
|  | 1F-3/ CGAT | 0.346/ 0.290 | 177:512/ 76:262 | 2.4 | 0.12 | | |
|  | 2-2/ ATAT | 0.063/ 0.088 | 32:512/ 23:262 | 1.68 | 0.20 | | |

## **Table S2. Univariable and multivariable logistic regression analysis to assess the association between 25(OH) D and DBP levels and risk of disease development, adjusted for haplotype or diplotype in males and females.**

|  | **Male** | | | |  | **Female** | | | |
| --- | --- | --- | --- | --- | --- | --- | --- | --- | --- |
|  | **Univariable** | | **Multivariable^†^** | |  | **Univariable** | | **Multivariable^†^** | |
|  | OR (95%CI) | p-value | OR (95%CI) | p-value |  | OR (95%CI) | p-value | OR (95%CI) | p-value |
| **Total 25(OH) D, (nmol/L)** | 1.01 (0.99-1.02) | 0.45 | 1.00 (0.99-1.02) | 0.52 |  | 0.99 (0.98-1) | 0.243 | 0.99 (0.99-1.00) | 0.18 |
| **DBP (mg/L)** | 1.00 (1.00-1.00) | 0.31 | 1.00 (1.00-1.00) | 0.50 |  | 1.00 (1.00-1.00) | 0.433 | 1.00 (1.00-1.00) | 0.07 |
| **Haplotype** |  |  |  |  |  |  |  |  |  |
| CT | ref. | - | ref. | - |  | ref. | - | ref. | - |
| CG | 0.91 (0.48-1.71) | 0.76 | 1.26 (0.61-2.63) | 0.53 |  | 1.08 (0.73-1.59) | 0.72 | 1.06 (0.66-1.72) | 0.80 |
| AT | 0.64 (0.32-1.28) | 0.21 | 0.81 (0.38-1.7) | 0.58 |  | 1.29 (0.83-1.99) | 0.26 | 1.33 (0.82-2.16) | 0.26 |
| **Total 25(OH) D, (nmol/L)** | 1.01 (0.99-1.02) | 0.45 | 1 (0.98-1.02) | 0.80 |  | 0.99 (0.98-1) | 0.243 | 1 (0.98-1.01) | 0.43 |
| **DBP (mg/L)** | 1.00 (1.00-1.00) | 0.31 | 1 (0.99-1.00) | 0.20 |  | 1.00 (1.00-1.00) | 0.433 | 1 (1-1) | 0.17 |
| **Diplotype** |  |  |  |  |  |  |  |  |  |
| CTCT | ref. | - | ref. | - |  | ref. | - | ref. | - |
| CGCT | 7.14 (0.56-90.8) | 0.13 | 13.86 (0.97-198.65) | 0.05 |  | 0.46 (0.11-1.85) | 0.27 | 0.4 (0.09-1.81) | 0.24 |
| CTAT | 4.4 (0.32-60.61) | 0.27 | 4.9 (0.34-70.11) | 0.24 |  | 0.67 (0.15-2.93) | 0.59 | 0.6 (0.13-2.87) | 0.53 |
| CGCG | 4.32 (0.37-49.9) | 0.24 | 10.97 (0.75-160.96) | 0.08 |  | 0.58 (0.15-2.2) | 0.42 | 0.49 (0.11-2.27) | 0.36 |
| CGAT | 3.6 (0.31-41.37) | 0.30 | 8.24 (0.64-106.29) | 0.11 |  | 0.89 (0.23-3.44) | 0.87 | 0.79 (0.18-3.43) | 0.75 |
| ATAT | 1.43 (0.1-20.44) | 0.79 | 1.91 (0.13-28.4) | 0.64 |  | 0.56 (0.13-2.39) | 0.44 | 0.61 (0.13-2.86) | 0.53 |
| †Adjusted for BMI, Sampling time of year (dark/light), Smoking ever, Educational level (academic/ no academic), age at the time of sampling | | | | | | | | | |
